# Neural Network for Enhancing Microscopic Resolution Based on Images from Scanning Electron Microscope

**DOI:** 10.3390/s21062139

**Published:** 2021-03-18

**Authors:** Chia-Hung Dylan Tsai, Chia-Hao Yeh

**Affiliations:** Department of Mechanical Engineering, National Yang Ming Chiao Tung University, Hsinchu 30010, Taiwan; a0917349016.me06g@nctu.edu.tw

**Keywords:** image enhancement, microscope, microfluidics, artificial neural network

## Abstract

In this paper, an artificial neural network is applied for enhancing the resolution of images from an optical microscope based on a network trained with the images acquired from a scanning electron microscope. The resolution of microscopic images is important in various fields, especially for microfluidics because the measurements, such as the dimension of channels and cells, largely rely on visual information. The proposed method is experimentally validated with microfluidic structure. The images of structural edges from the optical microscope are blurred due to optical effects while the images from the scanning electron microscope are sharp and clear. Intensity profiles perpendicular to the edges and the corresponding edge positions determined by the scanning electron microscope images are plugged in a neural network as the input features and the output target, respectively. According to the results, the blurry edges of the microstructure in optical images can be successfully enhanced. The average error between the predicted channel position and ground truth is around 328 nanometers. The effects of the feature length are discussed. The proposed method is expected to significantly contribute to microfluidic applications, such as on-chip cell evaluation.

## 1. Introduction

The optical microscope (OM) is one of the most important inventions in the 17th century. It has been used in different fields, such as in medicine, engineering, biology, astronomy, etc. [1,2]. The resolution of a microscopic image is important particularly when it comes to evaluating cell properties on a chip [3,4,5]. For example, Tsai et al. [6] evaluate cell deformability based on cell speed passing through a constriction channel, and they estimate the dimension of the channel by the cross-reference between OM image and a laser microscope. However, the optical resolution is limited due to different optical effects. For example, the wavelength of the visible lights, as Ernst Abbe [7,8] first demonstrated the limitation of microscopic images in 1873. The limit of optical resolution was later defined by diffraction limit that the minimum distinguishable distance of two dots is around 200 nm [9,10]. Liu et al. [11] revealed the effects of light scattering on optical resolution by employing the hyperboloid focusing method in the Monte Carlo simulation for photoacoustic microscopy. They discussed the relation between the lateral resolution and the depth of focal plane that the resolution would quickly degrade when the depth of focal plane is greater than a threshold.

To cope with the limits of microscopic resolution, different approaches for enhancing the resolution have been proposed and can be categorized into three categories, which are the approaches of hardware, software, and combination of both, respectively. For the enhancement methods using hardware, microscopes with working principles different from OM have been used for better image resolution. For example, Shiotari and Sugimoto [12] obtained ultrahigh-resolution imaging of water networks by an atomic force microscope (AFM). Scanning electron microscopy (SEM) is another tool for acquiring high-resolution images for micro/nano structures. In a SEM, electrons excited by an electron beam from a target surface are collected for reconstructing surface topography. The electrons are accelerated to high energies of between 2 keV and 1000 keV, corresponding to the wavelengths of 0.027 nm and 0.0009 nm, respectively [13]. Due to such a short wavelength, the resolution of a SEM can be greatly beyond the optical limits of visible lights. 

For the enhancement methods using software, image processing algorithms have been used for the enhancement. For example, Thévenaz et al. [14] introduce nearest-neighbor interpolation, linear interpolation, etc., for resampling images for better resolution. Walker enhanced the optical imaging by using an iterative spectral extrapolation algorithm and achieved resolution beyond the Rayleigh criterion [15]. Fattal proposed an edge-frame continuity modulus for upsampling the low-resolution images [16]. Artificial intelligence (AI) has also been an emerging tool for enhancing the resolution of images [9]. For example, Wang et al. [17] proposed a convolutional neural network (CNN) method to reconstruct high-resolution porous structures based on computed tomography from low-resolution images. Dong et al. [18] achieved super-resolution images by directly learning the mapping between low and high resolution images with a deep CNN. Dong et al. [19] also proposed an hourglass-shape CNN for accelerating the calculation of super-resolution. Song et al. [20] applied deep CNN to the depth of the images for depth super-resolution.

For the enhancement method using both hardware and software, the most famous one should be 2014 Nobel Prize Laureates, Betzig, Hell and Moerner, who incorporated the manipulation of fluorescent molecules in microscopy and achieved super-resolution microscopy [21]. Holography, which is a technique to reconstruct 3D real-world images from diffraction signals, has also been used for enhancing optical resolution. For example, Eom and Moon [22] utilize inline hologram reconstruction to achieve 3D high-resolution images. Huang et al. [23] also employ holographic technology to enhance the resolution for a lens-less on-chip microscope.

**Figure 1:**
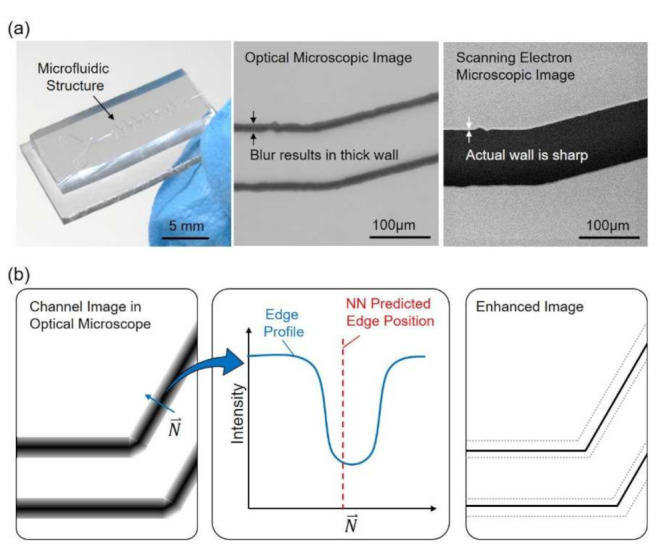
An overview of the proposed method for enhancing the resolution of an optical microscope (OM). (**a**) A microfluidic chip and its structure observed with an OM and a scanning electron microscopy (SEM). (**b**) The edges of the structure are in bold lines due to the optical effects. Artificial neural network is applied to predict the edge position and to enhance the image.

Figure 1 shows an overview of the proposed method, which focuses on enhancing the resolution of microscopic images with an artificial neural network (NN) and reference images acquired from a SEM. An example of a microfluidic chip along with a part of microstructure taken by an OM and a SEM is shown in Figure 1a, from the left to the right, respectively. It can be found that the channel walls are in bold and dark lines in the OM image while the same wall is actually sharp in the SEM image. The presentation of bold lines for the channel walls is not necessarily caused by the optical limits but also many other optical effects, such as diffraction of light and scattering due to surface roughness. An illustrative sample of channel image and intensity profile are shown in Figure 1b. The intensity profile along the direction N, perpendicular to the edge, is illustrated in the middle of Figure 1b. The prediction of edge position is calculated from the profile through a trained NN, and is labeled as NN predicted edge position in Figure 1b. The rightmost illustration in Figure 1b shows that the image is enhanced based on the prediction and the channel wall is enhanced with a sharp edge after the process.

To the best of the authors’ knowledge, this paper is the first work trying to enhance the resolution of OM images using the neural network with corresponding images from a SEM. The method provides not only the enhancement of the image resolution, but also incorporates physical meanings of the results based on actual measurement from a SEM. 

## 2. Materials and Methods

### 2.1. Overview of the Proposed Method

**Figure 2:**
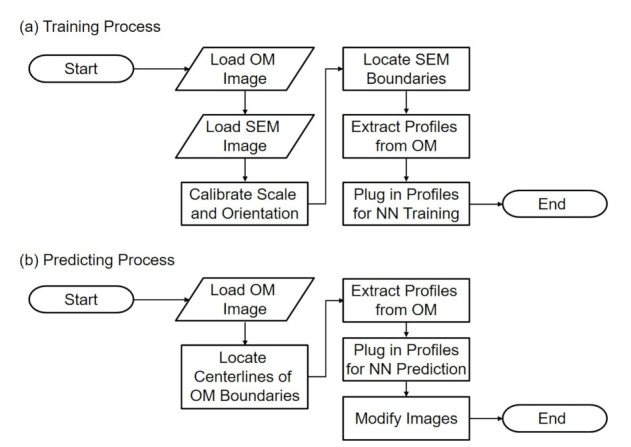
The flowcharts of the proposed method of (**a**) training process and (**b**) predicting process.

Figure 2a,b shows the flowcharts of training and predicting in the proposed method. During the training, images from the OM and SEM are first acquired and loaded to the program. Since the images are taken from two independent instruments, the calibrations of scales and orientations are necessary and important for determining the correlation between the paired images. After the scaling and alignment, the edge of SEM images is located with image processing algorithms, such as canny method [24]. The intensity profiles perpendicular to the obtained edge in the corresponding OM images are extracted for the input features in the NN. Finally the profiles and the location of the SEM edge, the ground truth, are plugged into the NN for network training. The flowchart in Figure 2a will result in a trained NN. 

During the prediction as shown in Figure 2b, the trained network is used to predict the location of the actual edge from a blurry edge in an OM image. Instead of locating SEM boundaries, the centerlines of the boundaries of OM images are located using image processing methods. The intensity profiles on the OM centerlines along the direction perpendicular to the centerlines are extracted from OM images, and are plugged in the trained NN for prediction. The last step of prediction is to modify the OM image based on the prediction results. 

### 2.2. Experimental Setup

**Figure 3:**
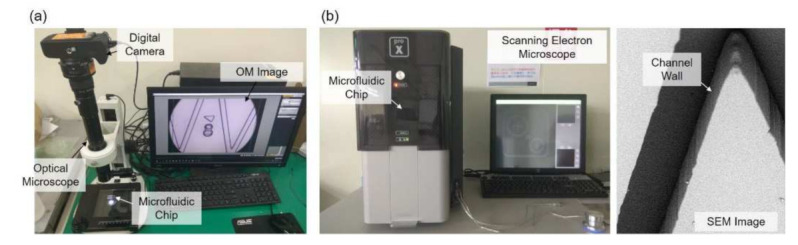
Microscopes for obtaining images. (**a**) Optical microscope. (**b**) Scanning electron microscope and a sample image taken from it.

The experimental setup is shown in Figure 3, where Figure 3a,b are the OM and SEM systems, respectively. The OM system is constructed with an OM, a digital camera, and a PC as shown in Figure 3a. The target microfluidic channel is placed under the lens for observation. The SEM system includes a desktop SEM (Phenom G2 Pro, Thermo Fisher Scientific Inc., Waltham, MA, USA) and a sputter coater (Cressington 7002, TED PELLA Inc., Redding, CA, USA). The microstructure on the chip is first coated with a thin layer of gold with the sputter coater and then put onto a special holder for being observed in the SEM. A SEM image with the chip tilted with an angle of 45 degree is shown in Figure 3b, and it shows that the channel walls are almost perpendicular to the chip base. 

### 2.3. Target Channel

**Figure 4:**
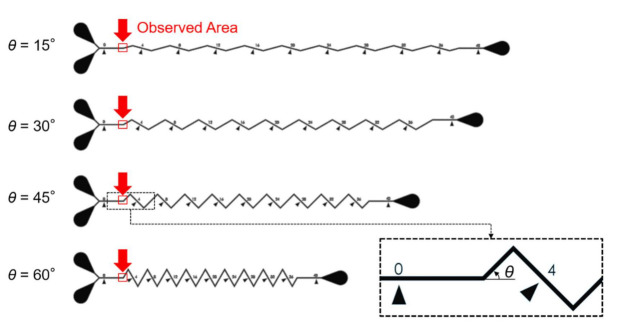
The design of the microfluidic chip and the chosen locations for machine learning.

Microfluidic channels used for on-chip mixing, as the design shows in Figure 4, are employed as target channels in this paper [25]. The channels are on a microfluidic chip made of polydimethylsiloxane (PDMS). The chip is cured in an oven at 95 ℃ for 40 min from a mixture of PDMS gel and curing agent (DC184, Dow Corning, Midland, TX, USA) at the ratio of 10:1. The observation targets are chosen as the starting regions of the zigzag channels, which include four different zigzag angles. The microchannels in Figure 4 were designed for investigating the mixing performance of zigzag channel, and the dimensions of the channels are particularly important because it would change fluidic dynamics. Therefore, the proposed method is expected to contribute to realizing the actual dimension from the blurry images of the microstructures. 

### 2.4. Image Pre-Processing and Calibration

Because images are taken from two different microscopes, an OM and a SEM, it is important to perform pre-processing on the images, so their scale, position, and orientation can be correctly aligned for later learning with NN. The scaling of the two images are done by a scaling factor of calibration

(1) $$C_{cali}=\frac{C_{OM}}{C_{SEM}}$$

where $C_{cali}$, $C_{OM}$, and $C_{SEM}$ are the scaling factors for calibration, *OM* images, and *SEM* images, respectively. While $C_{OM}$ and $C_{SEM}$ are determined based on the settings on the microscopes and are in the unit of μm per pixel, $C_{cali}$ is calculated for resizing OM images. The resizing is performed with “nearest” algorithm using image processing toolbox in Matlab (R2019a, The MathWorks, Inc., Natick, MA, USA). The positioning and rotating of the images are based on selected alignment reference in both *OM* and *SEM* images. In this paper, the centerlines along the microchannel are chosen as the reference for the position and orientation of the images. The goodness of alignment is evaluated with the sum of point-to-point distance between the two centerlines while the images are superimposed with different positions and orientations. The lowest value of the distance sum is considered as the best alignment of position and orientation for the paired images.

### 2.5. Feature Extraction and Neural Network

Feature extraction includes two main steps, locating the region of features and extracting features. In order to enhance the resolution of a given microstructure, the area around channel walls in the images are chosen as the region of interest, so that the blurry edges of the structure can be enhanced for a sharp edge. Image processing methods, such as Otsu method and canny method, in the image toolbox of Matlab are utilized for locating the region [26].

After the region of interest is located, the intensity profiles perpendicular to the edge are extracted as the features for the NN training. Since the gradient happens in the direction perpendicular to the channel walls, the direction is calculated using Sobel operator based in the directions along the intensity gradients. Two kernel matrices in the Sobel operator are [27]

(2) $$k_{x}=\left[\begin{array}{ccc} -1 & 0 & 1\\ -2 & 0 & 2\\ -1 & 0 & 1 \end{array}\right]\;\;\;k_{y}=\left[\begin{array}{ccc} 1 & 2 & 1\\ 0 & 0 & 0\\ -1 & -2 & -1 \end{array}\right]$$

where $k_{x}$ and $k_{y}$ are the kernels for calculating the derivatives in horizontal and vertical directions, respectively. The gradient profiles in the horizontal and vertical directions can be calculated with the kernels as

(3) $$G_{x}=k_{x}\times I\;\;\;G_{y}=k_{y}\times I$$

where I is the matrix of the image, and $G_{x}$ and $G_{y}$ are the intensity gradients of I along horizontal and vertical directions, respectively. The directions of intensity gradient on the given image can then be determined using

(4) $$\theta ={tan}^{-1}\frac{G_{y}}{G_{x}}$$

where θ is the direction of intensity gradient. Intensity profiles of length *L* from an edge point along the direction of θ are acquired as features. In order to increase the variety of the data, the portions of the intensity profile on the two sides of the edge point are randomly assigned, and it results in random target values, which are the positions of the edge point on the profiles. For example, the target value is said to be 0.5 if the intensity profile is acquired between the range of −0.5 L and 0.5 L from an edge point along the θ. The target value is said to be 0.1 if the intensity profile is acquired between the range of −0.1 L and 0.9 L from an edge point along the θ, and so on. The positive and negative directions are defined as the directions toward the outside and inside of the microfluidic channel, respectively. The intensity profiles and corresponding target values are then plugged into the NN machine for training.

**Figure 5:**
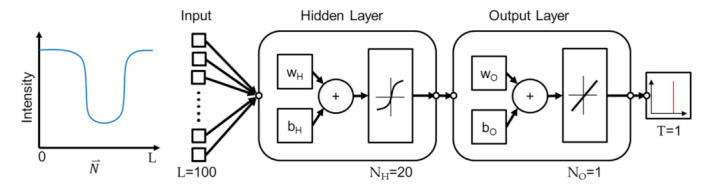
The structure of the neural network.

The proposed method employs a shallow network based on two considerations. First, the method is based on the observation and assumption that the real edge location is correlated with the intensity profile at an edge point along the direction perpendicular to the edge. Since the model is fairly simple, a shallow NN is believed being sufficient for predicting the real edge position from the profile. Second, while deep NN has advantages of realizing features, the intensity profile, into deeper levels and generally perform better than a shallow NN, the improvement is at the cost of computational complexity. A shallow NN is chosen in this work for better computational efficiency. Figure 5 illustrates the network for the NN learning using Matlab. A single hidden layer of network is employed. There are two layers in the network where sigmoid function and linear function are used for the outputs in the hidden and output layers, respectively. The features, which are intensity profiles, are transformed to the neurons with the activation function of sigmoid function in the first layer, where w_H_ and b_H_ are the weight and biases vectors.

Similar transformation is performed from the hidden layer to the output layer with a set of the weight vectors w_O_ and biases vectors b_O_, except the sigmoid function being replaced by a linear function. Finally, the prediction of wall position is generated from the output layer. The predicted output of the NN is a single number between 0 and 1, and the predicted number represents the location of the actual edge in the input vector. For example, if the predicted number is 0.3 and the length of the intensity profile is 100, it indicates that the edge is at the location of 100×0.3=30 in the profile. To sum up, the network is trained with the locations of the edge in a microstructure and corresponding intensity profiles from an OM image. The trained network can be used to predict the edge position in an intensity profile.

The algorithm of Levenberg-Marquardt is used for training the NN [28]. All intensity profiles on the edges are divided into three groups for training, validating, and testing the neural network, and the data portions are 70%, 25%, and 5%, respectively. The sizes of the input length L, the neurons in the hidden layer and output layer, and the target length are specified as 100, 20, 1, and 1, respectively. The effect of different input lengths L will be discussed in the discussion section. The criteria for the completion of the training, is set as no decrease of validation error for six epochs, while the maximum of the epochs is set to 1000.

**Figure 6:**
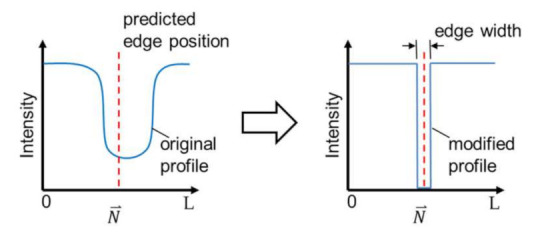
The image modification for sharpening the edge with a predicted edge position.

The last step of the enhancement is to modify intensity profiles in the OM images based on the prediction, so that the bold channel walls become a sharp edge in an enhanced image. Figure 6 illustrates an example of the modification method. The modification is done by replacing the intensity profiles with values based on the predicted edge position. The elements of the profile at the predicted edge position with a specified edge width are filled with the value of zero while the rest is filled with the value at the ends of the original profile. The edge width, as indicated in Figure 6, is set to 5 pixels in this work. The width of 5 pixels is for a better presentation in the enhanced image since the unit width of 1 pixel would result in a very thin line in the image. Finally, the original intensity profile is replaced by the modified intensity profile at every single edge point in the region of the interest in OM images along the direction perpendicular to the edge, and the enhancement is completed.

## 3. Results

The proposed method is validated with the design of microstructures shown in Figure 4 with images taken by an OM and a SEM. The results of each processing steps are presented as follows.

### 3.1. Scaling and Alignment of Images from OM and SEM

**Figure 7:**
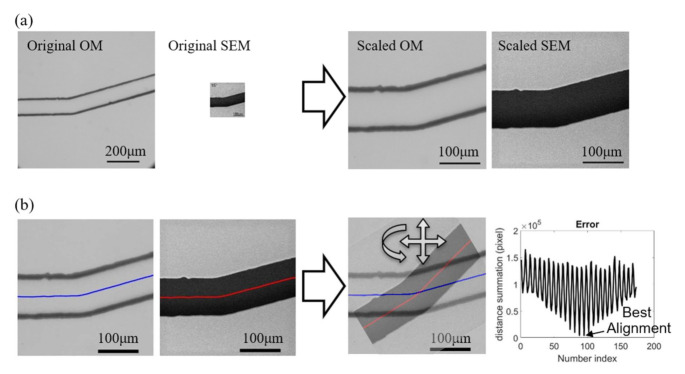
Preprocess for image calibrations. (**a**) Scaling. (**b**) Positioning and rotation.

Figure 7 demonstrates the pre-process with an example of paired images from the OM and SEM. The scales of the OM and SEM in the used instruments and the settings are $C_{\mathrm{OM}}=0.212$ [μm/pixel] and $C_{\mathrm{SEM}}=0.328\;[\mu m/$pixel], respectively. Figure 7a shows the images before and after the scaling. According to the images, it can be found that the OM has a larger viewing window than SEM while its digital resolution is also slightly higher than SEM owing to the high-resolution charge-coupled device (CCD) in the mounted camera (EOS m50, Canon Inc., Tokyo, Japan). However, the actual image resolution of OM is not as good as SEM, and it can be found from the scaled results on the right of Figure 7a that the edge of channel walls are in bold and dark lines in the scaled OM image while the edge is shaper in the SEM image. The spatial resolutions of both OM and SEM images are converted to the same value as $C_{\mathrm{OM}}=C_{\mathrm{SEM}}=0.328\;[\mu m/$pixel] after the scaling.

Figure 7b shows the process for aligning the two scaled images. The centerlines along the channel are first obtained as the reference for representing the positions and orientations of the images. The sum of point-to-point distance between the centerlines of two superimposed images are calculate while the images are adjusted with different positions and orientations, as shown on the right of Figure 7b. The right-most chart in Figure 7b shows an example of the calculated distance sum of the two centerlines with respect to the iterations of changing the position and orientation. The sinusoidal-like shape of distance sum is due to different orientations of the images while the low-frequency change is from the shifts of the position. The lowest value of the distance sum is determined as the position and orientation of the best alignment for the two images.

### 3.2. Feature Extractions

**Figure 8:**
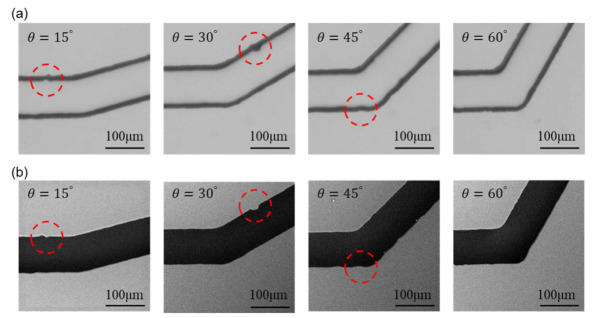
Scaled and aligned images from both microscopes with highlights on structural features. (**a**) Images from OM. (**b**) Images from SEM.

Figure 8 shows the images from OM and SEM after scaling and alignment. The images from the left to the right in Figure 8 are the channels with turning angles of $15^{o},\;30^{o},\;45^{o},$ and $60^{o}$, respectively. A few structural features, as non-smooth edges on the channel walls, are highlighted with red circles for demonstrating the detailed differences between OM and SEM images. The actual shape of such non-smooth microstructures cannot be clearly identified in the OM images because the channel walls are in bold lines while it can be better observed in the SEM images.

**Figure 9:**
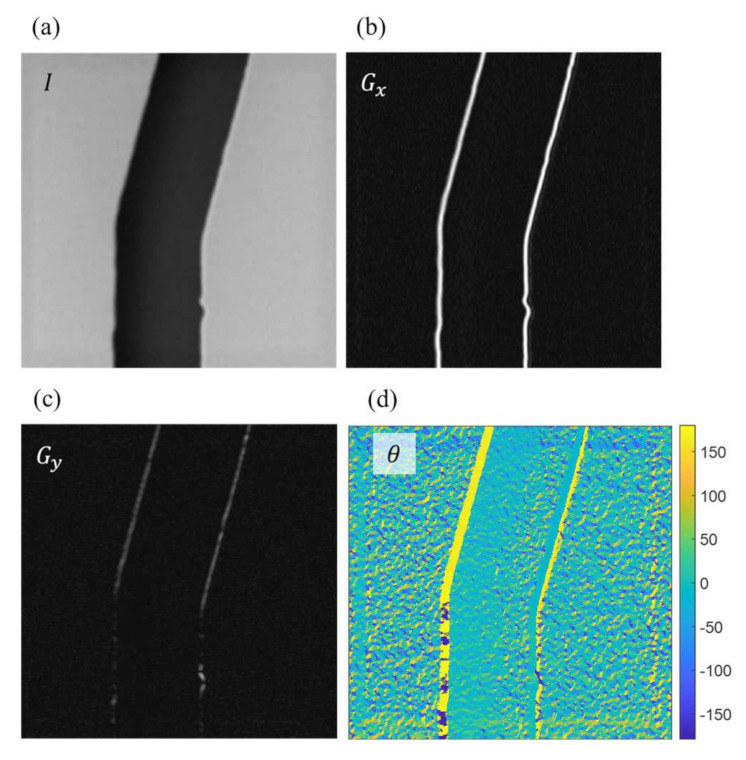
The directions perpendicular to the edge points are determined using Sobel operators. (**a**) Original image taken from the SEM. (**b**) The intensity gradients in the horizontal direction. (**c**) The intensity gradients in the vertical direction. (**d**) The directions of intensity gradients in degrees.

Figure 9 shows an example of determining the perpendicular directions at the edge points with Sobel operators. Figure 9a is an example of the SEM image. The intensity of the image is saved in the matrix of *I* with rows and columns equal to the length and width of the image. The intensity is the grayscale values of each pixel and ranged from 0 to 255 in an 8-bit image in this work. Figure 9b,c are the calculated results using Equation (2) with Sobel operator $k_{x}$ and $k_{y}$, and are the intensity gradients along the horizontal and vertical directions, respectively. Figure 9d is the determined direction using Equation (4) based on the crossing angles of the gradients in Figure 9b,c for each pixel in the image.

**Figure 10:**
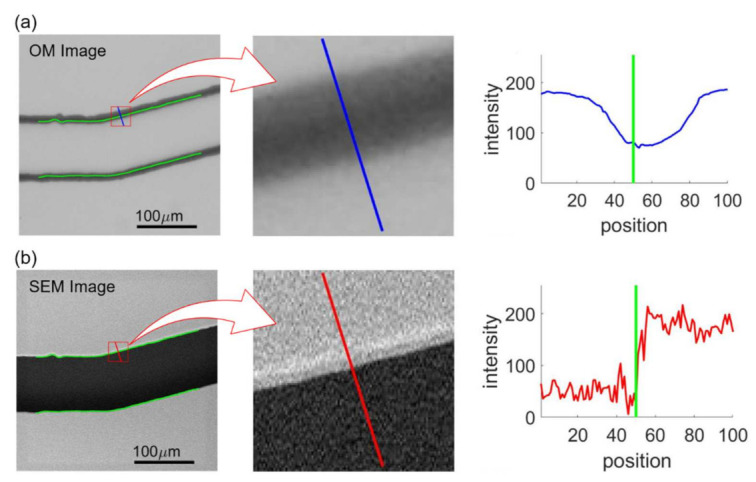
Intensity profiles in OM and SEM image samples. (**a**) OM image. (**b**) SEM image.

Figure 10 shows an example of feature extraction where Figure 10a,b shows the intensities at the same edge point from the aligned OM and SEM images, respectively. The green lines in the OM and SEM images in Figure 10 indicate the position of channel walls from the SEM image obtained with image processing. The perpendicular directions with respect to the wall are calculated based on the color gradients using Equations (2)–(4) and are shown as the blue and red lines in the middle of Figure 10a,b. The intensity profiles along the blue and red lines, whose lengths are specified as 100 pixels, are plotted on the right of Figure 10a,b, where the green lines indicate the detected edge position based on the SEM image. The intensity profiles of the OM image are used as input features for the NN training while normalized edge positions, which is 0.5 in the case of Figure 10b, determined from SEM image are used as the target values. Each edge point is sampled 9 times for different ratios of profile length toward inside and outside of the channel. The determined edge points are 1955, 2171, 2183, and 2063 for the channels with turning angles of $15^{o},\;30^{o},\;45^{o},\;$ and $60^{o}$, respectively. Therefore, the numbers of the training data are 17,595, 19,539, 19,647, 18,567 corresponding to the images of the turning angles of $15^{o},\;30^{o},\;45^{o}\;$, and $60^{o}$. For each image, at least 17,595 intensity profiles are extracted from the image and are plugged into the NN for the training.

### 3.3. NN Training with Images from OM and SEM

**Figure 11:**
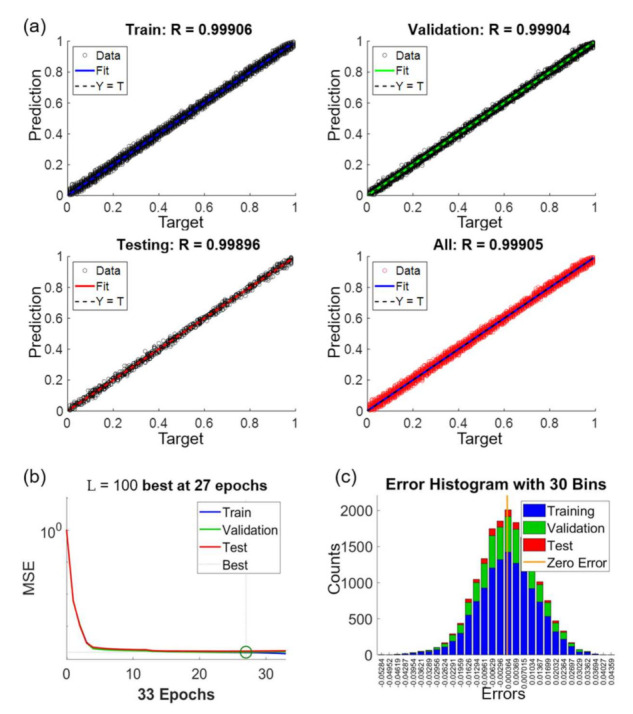
The performance of the network training for L = 100, N_H_ = 20 and $\theta =15^{{}^{\circ}}$. (**a**) Regression plots with different data sets. (**b**) Convergence of training. (**c**) Error histogram of the predictions.

Figure 11 shows the performance of the NN after the training. Figure 11a includes regression plots for the datasets of train, validation, test and all, as labeled on the charts. The x and y axes in Figure 11a are the given target values and the predicted values by the NN, respectively. For example, a target value of 0.3 indicates that the edge of an OM image is at the location of 30% with respect to the length of the intensity profile. The target values for the training are obtained from SEM images, which is used as the ground truth for the NN. On the other hand, the predicted values are calculated using the trained NN. According to the results, the coefficients of correlation are all above 0.99 for all the regression plots in the different datasets in Figure 11a. We would like to particularly note that the training for the NN is only based on the datasets of train and validation. The result of R = 0.99896 in the test dataset demonstrates that the NN can accurately predict the actual edge locations of microstructure from the given intensity profiles in OM images.

Figure 11b shows the convergence of the NN training. The x and y axes in the plots are the mean square error (MSE) and epoch, respectively. The value of MSE indicates the difference between the predicted value and target value. One epoch means one time of all the intensity profiles being processed in the NN. The criterion for the completion of the training, as explained in the section of method, is set as no decrease of validation error for six epochs. As the case shown in Figure 11b, it takes 33 epochs to reach to the criterion of training where the epoch of 27 is defined as the best fit.

Figure 11c shows the error histogram of the results between the given target values and predicted values. The error is distributed as a standard normal distribution and the error ranged from −0.055 to 0.045 in the normalized scale from 0 to 1. The zero of error is just located at the center of the distribution. The vertical axis shows the counts of the prediction error. The distributions of errors are similar in all three sets which indicate no sign of over-fitting of the neural network.

### 3.4. NN Prediction and Performance

The trained NN is used to predict edges from OM images without data from SEM. For enhancing the OM images, the regions for feature extraction are located by the detected channel walls in OM images. Because the channel walls are in thick lines in the OM images, the centerlines of the channel walls are selected as the region of extraction, and the intensity profiles perpendicular to the centerlines are extracted from the points on the centerlines as features. The features are plugged into the trained NN for the prediction.

**Figure 12:**
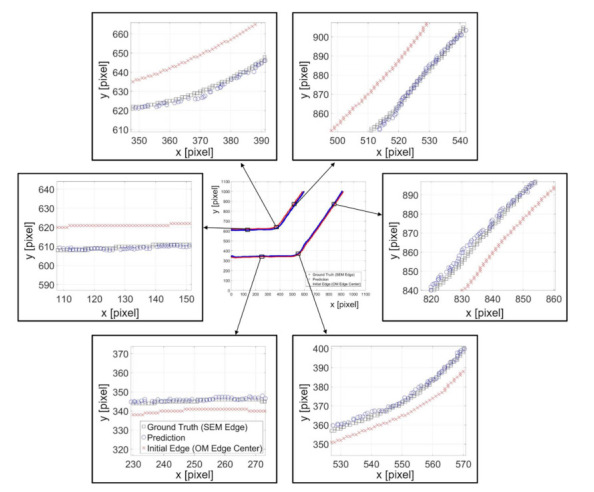
Examples of predicted edge position and the ground truth position from SEM images.

Figure 12 shows an example of predicted results with the channel angle of $60^{o}$. The feature length and neuron number in the hidden layer are 100 pixels and 20, respectively. The center of Figure 12 is the overview of the prediction where the marks of rectangles, circles, and crosses indicate the ground truth position from SEM edges, the predicted position from NN, and the center of the OM edges, respectively. Six randomly chosen locations on the edges are zoomed in for a better observation of the prediction performance. It can be found that the centerline, the red crosses, of the channel wall from OM images are around 15 pixels, which is approximately 5 micrometers, away from the SEM edges, the black boxes. The actual edges of the structure tend to lean to the center of the channel. The zoomed results in Figure 12 demonstrate that the proposed method can successfully predict the position of the edges based on intensity profiles on the edge of OM images, regardless the direction of the edge.

**Figure 13:**
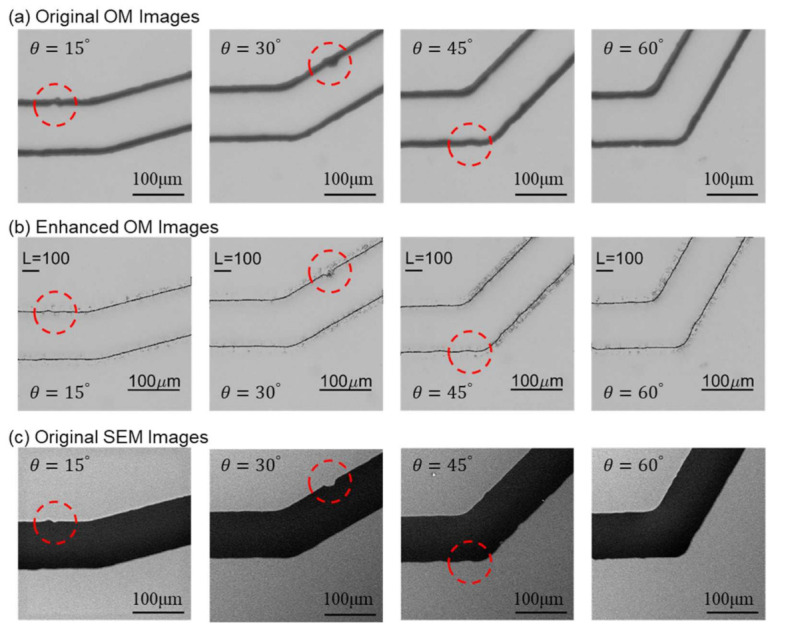
Comparison between original OM, enhanced OM, and SEM images.

Figure 13 shows the comparisons between original OM, enhanced images, and SEM images for the microfluidic channels with different turning angles. The original OM, enhanced images, and SEM images are shown in Figure 13a–c, respectively. The same structural features are circled as those in Figure 8. It can be seen that the enhanced OM images are very similar to the ones in the corresponding SEM images. For example, the circled area in the channel of $30^{o}$ is like an ink stain in the OM image in Figure 13a while it is actually a concave defect on the channel wall according to the SEM image, Figure 13c. The enhanced OM image in Figure 13b can successfully resemble the concave shape on the wall with NN prediction.

**Figure 14:**
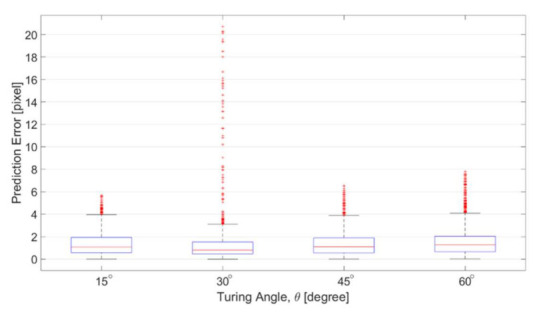
Prediction errors among the channels with different zigzag angles.

Figure 14 shows the errors of the prediction with respect to different turning angles. The prediction errors are defined as the distance between the predicted position and ground truth position from corresponding SEM images. The distance is calculated using quickhull algorithm for searching the nearest point in Matlab [29]. The results in Figure 14 are in the unit of pixels, which is approximately 0.328 micrometer per pixel. According to the results in Figure 14, the average of the prediction errors among different angles are all around 1 pixel, and the distribution is focused in the area between 0 and 4 pixels. Red dots in Figure 14 are the outliers based on the overall data distribution. The outlier is defined as the errors greater than 1.5 times of the interquartile range, which is the distribution width between 25% and 75% of the data. The prediction error is fairly small for the channels of four turning angles. Although the number of outliers is slightly greater in the channel of 30 degree, no significant difference was found between different angles, which indicates that the proposed method is applicable to the channel with different turning angles.

## 4. Discussions

The length of intensity profiles is labeled as L and is set as 100 pixel in the prior results. Different length of intensity profiles would greatly affect the training speed and might result in different NN predictions. Therefore, parameter study on L is performed with ten different lengths of L, and they are L = 10, 20, 30, 40, 50, 60, 70, 80, 90, and 100 pixels. For the example of L = 50, the intensity profile can be extracted from −25 to +25 pixel range from a given position in an OM image.

**Figure 15:**
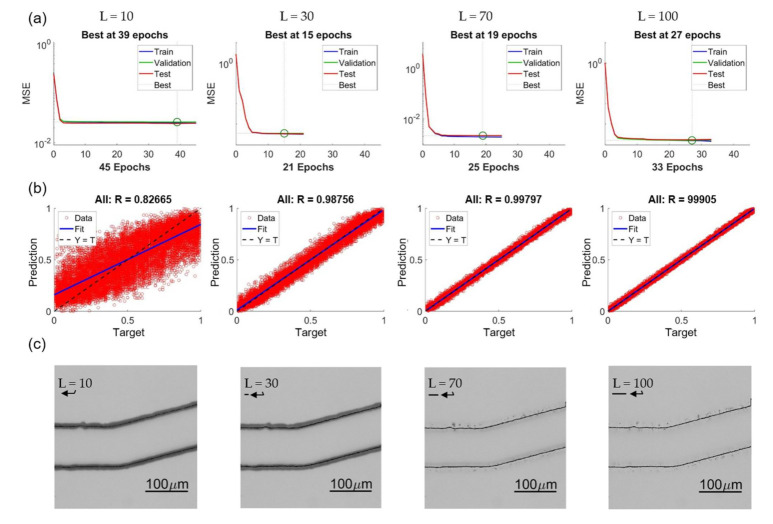
Effects of different feature lengths L. (**a**) Training convergence. (**b**) Regression plots. (**c**) Enhanced images.

Figure 15 shows four examples of training results with different lengths as L = 10, 30, 70, and 100. Figure 15a–c are the convergence of training, regression plot, and enhanced images, respectively. It is found that in the range of L = 10 to 100, the NN can always converge, and no clear trend of the epoch number is observed from the results in Figure 15a. The trend of the regression plots in Figure 15b is clear that the longer length L results in a better coefficient of correlation. We would like to specially note that the unit in the regression plot is normalized from 0 to 1, and it is not necessary that it directly represents the accuracy of the prediction in terms of pixel or micrometers. Figure 15c are the enhanced images modified from original OM images with predictions of different intensity lengths. The corresponding length of the intensity profiles are pointed by an arrow in each image in Figure 15c.

**Figure 16:**
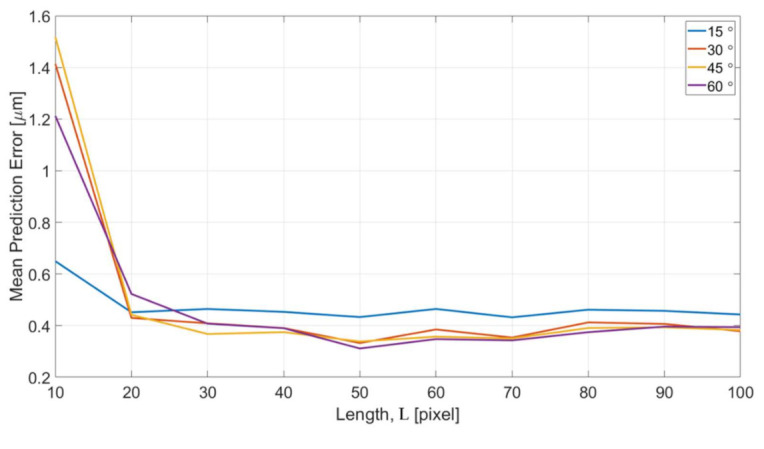
Effects of different feature length L.

Figure 16 shows the error of different length of intensity profiles between the predicted edge position from OM images and actual edge positions determined from SEM images. The x and y axes are the specified length of intensity profile and the mean prediction errors, respectively. The error is converted to the unit of micrometers for a better understanding of the performance. According to the results in Figure 16, the trend of the error is consistent in that the errors converge with the increase of the length L for all four different turning angles of the channels. The error is around 0.4 micrometer which is approximately 1 pixel in the image after the convergence. The error is believed can be further reduced by training with a greater magnification ratio of SEM images.

The errors with L = 10 is found having significantly greater values than all other lengths. It can be interpreted based on the enhanced images and the represented bar of length L in Figure 15c. The length of 10 pixel is too short and it is difficult to predict a position outside the range of the profile. According to the visual observation on the images of OM and SEM, the distance between the edge position from OM images and SEM images is around 10–15 pixels. Therefore, it is outside of the +5 to −5 range for the training length of 10 pixels. In other words, in order to properly predict the edge position of the SEM images from the OM images, a length of 20 is needed, and it well matched to the results shown in Figure 16.

**Figure 17:**
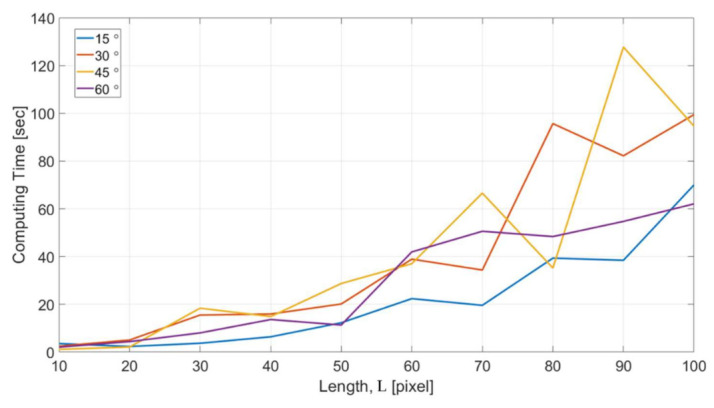
The computational cost with different feature length.

Figure 17 shows the computational cost with respect to different feature length L for the microstructures with four different turning angles. The computing time in the y axis indicates the time for NN to reach the completion of the training for all the dataset acquired in one single OM image. The results show a consistent trend that the computing time increases with the increase of feature length. For the feature length of 100 pixels, it took around 1–2 min for handling all the data. The optimized length for the intensity profiles can be decided based on the consideration of both the results in Figure 16 and Figure 17.

The proposed method aims to find the relation between the intensity profile of blurred edges in an OM image and the actual edge position detected from a SEM image. In the case of microfluidic images in the paper, the actual edges on the profiles are not at the center of the profile but tend to be leaner to the center of the channel. The trend may, or may not, be true when it comes to a different tapered structure or using a different microscope. In other words, microfluidic images are just examples for validating the proposed method and the trained network is only applicable for the setup. The proposed method is expected to be able to apply to different objects, such as microstructure made of different materials and biological cells/tissues. The relation between the intensity profile and the actual structure on different setups and targets needs to be trained before use. Furthermore, there is no need to acquiring a large amount of OM and SEM images for training the proposed method. The input features and target values for the NN are the intensity profiles along the edges in OM images and detected edges in corresponding SEM images. A large number of intensity profiles can be acquired from one single set of OM and SEM images. Take the OM and SEM data in the paper as an example, more than 17,595 feature profiles can be extracted from one single set of images, and according to the results, the feature size is sufficient for the training. For practical application, the proposed method can be used as a calibration process to a microscope before use.

## 5. Conclusions

A NN method for improving the resolution of images from an OM based on a SEM is proposed and experimentally tested. According to the results, the proposed method successfully enhances the OM image of microstructure with different turning angles on a microfluidic chip. After the enhancement, the blurred edges become sharp and well matching to the edge positions in the corresponding SEM images. The mean prediction error is about 1 pixel, and is approximately 0.328 micrometers. The error is possible to be further reduced if SEM images with higher resolution are taken for the training. Different lengths of intensity profiles perpendicular to the edges of the structure are tested and discussed. The results show that the errors converge with the increase of the length L. The minimum required length of intensity profile is 20 pixels in the case, and is generally based on the distance between the point of feature extraction and the actual edge position. The proposed method provides enhanced resolution for measuring the dimensions of microstructure or cells using an OM. Such an enhancement can contribute to on-chip cell evaluation and other researches involving an OM.
